# Effect of inhalation drug therapy on inflammatory factors and quality of life on stable chronic obstructive pulmonary disease

**DOI:** 10.12669/pjms.40.7.7106

**Published:** 2024-08

**Authors:** Guang-ming Dai, Hong Wang

**Affiliations:** 1 Guang-ming Dai Department of Geriatrics, First People’s Hospital of Suining City, 629000 Suining, Sichuan, China; 2 Hong Wang Health management Center, West China Hospital, Sichuan University, Chengdu 610041, Sichuan China

**Keywords:** COPD, Pulmonary function, Inflammatory markers, Quality of life

## Abstract

**Objective:**

To investigate the clinical efficacy of inhaled triple therapy in the treatment of stable chronic obstructive pulmonary disease (COPD).

**Methods:**

This is a clinical comparative study. A total of 80 patients with COPD admitted to the First People’s Hospital of Suining City from June 2020 to June 2023 were included and randomly divided into the study (conventional COPD treatment + inhaled triple therapy) and control (conventional COPD treatment) groups. The clinical efficacy of inhaled triple therapy and adverse reactions of the two groups to the treatment were observed. Clinical efficacy was assessed through changes in pulmonary function indexes, and comparisons of T lymphocyte subsets and serum inflammatory markers were conducted. In addition, St George’s Respiratory Questionnaire (SGRQ) was employed for the quality-of-life assessment.

**Results:**

The study group showed a significantly higher total efficacy than the control group (*P* < 0.05), with no significant difference in terms of adverse reactions between them (*P* > 0.05). After treatment, the study group showed better improvement in pulmonary function indexes, such as forced expiratory volume in one second (FEV_1_), FEV_1_ as a percentage of the expected value, forced vital capacity (FVC) and FEV_1_/FVC, compared with the control group (all *P* < 0.05). In addition, the study group presented higher levels of T lymphocyte subsets CD3^+^, CD4^+^ and CD4^+^/CD8^+^ than the control group(all *P* < 0.05). After treatment, the levels of inflammatory markers tumour necrosis factor-α, leukotriene B4 LTB4 and interleukin-6 in the study group decreased more than those in the control group (all *P* < 0.05). Moreover, the study group attained a lower SGRQ score than the control group (all *P* < 0.05).

**Conclusion:**

Triple inhalants further improve the clinical efficacy of the treatment of COPD.

## INTRODUCTION

Chronic obstructive pulmonary disease (COPD) refers to a chronic airway inflammatory reactive respiratory disease characterised by persistent airflow limitation; this condition shows a close bearing on the aggravation of the body’s inflammatory response and expressions of inflammatory mediators.^1^-^4^ COPD primarily influences the lungs but can also cause extrapulmonary adverse effects. The main clinical manifestations of COPD include cough, wheezing and expectoration, and the disease may further develop into pulmonary hypertension, chronic pulmonary heart disease and respiratory failure in case of poor management.^5^,^6^ The elderly population shows higher vulnerability to COPD compared with young populations. With population ageing and the aggravation of environmental pollution, the number of patients with COPD is increasing year by year. The World Health Organization predicts a continued rise in the prevalence of COPD in the next 40 years; by 2060, more than 5.4 million people are expected to die annually from COPD and related diseases.^7^-^9^ COPD is characterised by its slow progress and frequent exacerbations.

COPD features a very high morbidity and mortality rate, seriously affecting the life safety of patients. The pathogenesis of COPD involves major inflammatory factors, including tumour necrosis factor-α (TNF-α), leukotriene B4 (LTB4) and interleukin-6 (IL-6).^10^,^11^ A variety of treatments are available for COPD, but the prognosis in the majority of cases remains suboptimal in terms, and suffer in terms of physical and mental health, resulting in their reduced quality of life. Thus, active and effective intervention and treatment need to be carried out to relieve the clinical symptoms and improve the quality of life of patients with COPD. For acute exacerbation of COPD, antispasmodic (such as salbutamol, terbutaline and salmeterol), antiasthmatic and anti-infective disease controls are mostly adopted, and for stable COPD, priority is given to drug intervention to relieve chronic airway inflammatory response to relieve the symptoms of patients and reduce acute exacerbations. This study aimed to investigate the effect of inhaled triple therapy on inflammatory factors and the quality of life of patients with stable COPD.

## METHODS

This research is a clinical comparative study. A total of 80 patients with COPD admitted to the First People’s Hospital of Suining City from June 2020 to June 2023 were ncluded and randomly divided into observation and control groups via the random number method, with 40 cases in each group. The observation group comprised 21 males and 19 females, aged 39–78 (55.58 ± 8.61) years, with a disease course of 4–12 (6.33 ± 1.65) years. The control group included 23 males and 17 females, aged 39–76 (56.85 ± 8.08) years, with a disease course of 3–11 (6.60 ± 1.75) years.

### Ethical Approval:

The study was approved by the Institutional Ethics Committee of First People’s Hospital of Suining City (No: 2023(1); Date: January 17, 2023), and written informed consent was obtained from all participants. No statistically significant difference was observed in the comparison of general data, although they were comparable between the two groups (*P* > 0.05; Table-I).

**Table-I:** Comparative analysis of the general data of the two groups.

Group	n	Age (years old) (*χ̅*±*S*)	Gender (Male/Female, n)	BMI (kg/m^2^) (*χ̅*±*S*)	Smoking (Yes/No, n)	Disease duration (years) (*χ̅*±*S*)
Observation group	40	55.58±8.61	21/19	25.85±2.14	25/15	6.33±1.65
Control group	40	56.85±8.08	23/17	26.23±2.31	26/14	6.60±1.75
*χ^2^*		0.683	0.202	0.752	0.054	0.722
*P*		0.497	0.653	0.454	0.816	0.473

### Inclusion criteria:

Patients who met the diagnostic criteria formulated by the Global Initiative for Chronic Obstructive Lung Disease (GOLD)^12^ were included in the study:

- A postbronchodilator forced expiratory volume in one second (FEV1)/forced vital capacity (FVC) ratio of less than 0.70, which ‘confirms the presence of persistent airflow limitation’;
- appropriate symptoms’, including dyspnoea, chronic cough, sputum production or wheezing;
- Significant exposures to noxious stimuli’, such as a history of smoking cigarettes or other environmental exposures.
- Patients who fit the Group D profile, i.e. patients with frequent (≥2) outpatient exacerbations or one or more hospitalisations, in the symptom- and outcome-oriented pharmacotherapy model proposed in the GOLD 2018 document. 12
- Patients with clear awareness and understanding of the study and who agreed and signed the study consent form.

### Exclusion criteria:

- Pregnant or lactating women.
- Patients with mental illness or cognitive impairment.
- Patients with central nervous system diseases.
- Patients with severe liver and kidney function injury and those who are allergic to the drug used in this study.
- Patients already on bronchodilator therapy, malignancy and bronchiectasis.
- Patients with bronchial asthma, pneumonia and other lung diseases.

All enrolled patients were subjected to routine anti-inflammation treatment, asthmatic relief, spasmodic, expectorant and other symptomatic support, provided with psychological adjustment, dietary guidance and oxygen inhalation and encouraged to quit smoking and start exercising. Based on the above treatment, the control patients received long-acting beta-agonist (LABA)/inhaled corticosteroid (ICS) inhalation therapy (50 μg salmeterol, 250 μg fluticasone propionate). Those in the study group were treated with triple therapy (100 μg fluticasone furoate, 62.5-μg umeclidinium bromide and 25-μg vilanterol trifenatate) in addition to the treatment in the control group, once a day, with one inhalation each time. Both groups of patients underwent a three-month treatment cycle.

### Observation indexes:

- The following pulmonary function indexes were detected in the two groups before and after treatment: forced expiratory volume in one second (FEV_1_), FEV_1_ as a percentage of the expected value (FEV_1_%), FVC and FEV_1_/FVC levels.
- The elbow venous blood of the two groups was collected before and after treatment, and the levels of CD3^+^, CD4^+^, CD8^+^ and CD4^+^/CD8^+^ of T lymphocyte subsets were compared.
- The changes in serum inflammatory factors TNF-α, LTB4 and IL-6 before and after treatment were compared between the two groups.
- The St. George’s Respiratory Questionnaire (SGRQ) was used to evaluate the quality of life of the patients, including symptoms, activities and the impact on daily activities. The questionnaire comprises 50 questions focusing on symptoms such as asthma and cough. The activities included climbing, dressing and housework. Effects on daily activities comprised insecurity, disappointment, anxiety and impaired social interaction. The higher the score, the poorer the quality of life.
- Clinical efficacy and adverse reactions during treatment of the two groups were observed. Efficacy evaluation criteria.

### Markedly effective:

The efficacy evaluation criteria included relief of symptoms (e.g. cough, expectoration, shortness of breath and wheezing) and disappearance of lung rales. Effective: The results on the effectiveness of the proposed treatment consisted of the significantly improved above symptoms of the patients and pulmonary rales. Invalid: The invalid result is the non-significant improvement in the clinical symptoms and signs of the patient. In addition, auscultation failed to attenuate the pulmonary rales. Total efficacy = (markedly effective + effective)/total number of cases × 100%. The adverse reactions included throat discomfort, nausea, hoarseness and dry mouth.

### Statistical Analysis:

Statistical analyses of all data in this study were conducted using SPSS22.0, and measurement data were expressed as mean ± standard deviation (*χ̅*±*S*). T-test was used in the comparison of results before and after treatment. The power of the test/confidence interval was 95%, enumeration data were expressed as n (%), and comparison between groups was performed via the χ² test. *P* < 0.05 indicates a statistically significant difference.

## RESULTS

Comparison between the two groups before treatment showed no statistically significant difference (*P* > 0.05). After treatment, significant improvements were noted in the FEV_1_, FEV_1_%, FVC and FEV_1_/FVC of the two groups, and the observation group exhibited a better improvement degree than the control group, with a statistically significant difference (*P* < 0.05; Table-II).

**Table-II:** Comparison of pulmonary function between the two groups (*χ̅*±*S*) n=40.

Observation index	Observation point	Observation group	Control group	t	p
FEV_1_ (L)	Before treatment	1.35±0.28	1.33±0.31	0.392	0.696
	After treatment*	2.19±0.25	1.70±0.24	8.677	0.000
FEV_1_%	Before treatment	48.68±3.67	48.06±4.21	0.430	0.668
	After treatment*	64.74±4.58	56.55±5.07	7.579	0.000
FVC (L)	Before treatment	2.28±0.65	2.26±0.73	0.129	0.898
	After treatment	2.87±0.63	2.51±0.50	2.820	0.006
FEV_1_/FVC	Before treatment	0.64±0.23	0.65±0.26	0.206	0.838
	After treatment*	0.79±0.17	0.70±0.13	2.835	0.006

***Note:***

* P<0.05 compared with the same group before treatment.

Before treatment, statistically significant differences were revealed by the levels of CD3^+^, CD4^+^, CD8^+^ and CD4^+^/CD8^+^ in the two groups were compared (*P* > 0.05). After treatment, the levels of CD3^+^, CD4^+^, CD4^+^/CD8^+^ in the two groups were better than those before treatment. In addition, the observation group exhibited an increase higher than that in the control group, with statistically significant differences (*P* < 0.05). No significant difference was observed in the changes in CD8^+^ before and after treatment of the two groups (*P* = 0.277, *P* = 0.254; Table-III).

**Table-III:** Comparison of T lymphocyte subsets levels in the two groups (*χ̅*±*S*) n=40.

Observation index	Observation point	Observation group	Control group	t	p
CD3^+^ (%)	Before treatment	42.18±2.06	42.13±1.32	0.129	0.897
	After treatment*	48.78±1.19	45.34±1.60	10.901	0.000
CD4^+^ (%)	Before treatment	24.73±3.63	25.43±3.93	0.824	0.412
	After treatment*	37.41±3.63	34.16±3.52	4.062	0.000
CD8^+^ (%)	Before treatment	21.40±3.77	22.29±3.50	1.094	0.277
	After treatment	22.16±3.76	23.09±3.51	1.150	0.254
CD4^+^/CD8^+^	Before treatment	1.17±0.14	1.16±0.21	0.224	0.823
	After treatment*	1.72±0.21	1.51±0.24	4.223	0.000

***Note:***

* *P*<0.05 compared with the same group before treatment.

Before treatment, comparisons were conducted on the serum levels of TNF-α, LTB4 and IL-6, with no statistically significant difference (*P* > 0.05). After treatment, significantly lower levels of TNF-α, LTB4 and IL-6 were in the two groups than those before treatment, and the observation group presented a greater degree of reduction than that in the control group, with a statistically significant difference (*P* < 0.05; Table-IV).

**Table-IV:** Comparison of inflammatory factor levels before and after treatment between the two groups (*χ̅*±*S*) n=40.

Group		Before treatment	After treatment	t	P
TNF-α (ng/L)	Observation group	45.98±7.21	21.25±5.31	17.463	0.000
	Control group	46.26±7.01	27.64±5.66	13.068	0.000
	*t*	0.175	5.205		
	*p*	0.861	0.000		
LTB4 (pg/L)	Observation group	52.26±16.84	15.46±4.52	14.131	0.000
	Control group	51.61±16.46	20.43±4.45	11.567	0.000
	*t*	0.180	4.949		
	*p*	0.857	0.000		
IL-6 (ng/L)	Observation group	18.83±6.05	8.55±2.29	10.049	0.00
	Control group	18.44±5.94	12.71±3.21	5.372	0.00
	*t*	0.285	6.672		
	*p*	0.776	0.000		

The comparison of SGRQ scores between the two groups before treatment revealed no statistically significant difference (*P* > 0.05). After treatment, the study group showed a lower SGRQ score than the control group, with a statistically significant difference (*P* < 0.05; Table-V).

**Table-V:** Comparison of SGRQ scores between the two groups (*χ̅*±*S*).

Group	n	Before treatment	After treatment
Observation group	40	68.88±4.46	33.75±4.38
Control group	40	68.05±3.10	38.08±3.11
*t*		0.960	5.090
*P*		0.340	0.000

The observation group exhibited a total effective rate of 97.50%, which was significantly higher than that of the control group (85.00%), with a statistically significant difference (*P* < 0.05; Table-VI). During treatment, adverse reactions occurred in both groups, but no statistically significant difference was observed (*P* > 0.05; Table-VII).

**Table-VI:** Comparison of clinical efficacy between the two groups [n (%)].

Group	n	Markedly effective	Effective	Invalid	Total effective rate (%)
Observation group	40	35 (87.50)	4 (10.00)	1 (2.50)	39 (97.50)
Control group	40	30 (75.00)	4 (10.00)	6 (15.00)	34 (85.00)
*χ*^2^ value					3.914
*P* value					0.048

**Table-VII:** Comparison of adverse reactions between the two groups after treatment [n (%)].

Group	n	Throat discomfort	Nausea	Hoarseness	Dry mouth	Incidence of adverse reactions
Observation group	40	2 (5.00%)	3 (7.50%)	0 (0.00%)	0 (0.00%)	5 (12.50%)
Control group	40	1 (2.50%)	1 (2.50%)	0 (0.00%)	1 (2.50%)	3 (7.50%)
*χ* ^2^						0.556
*p*						0.456

## DISCUSSION

As shown in this study, the two groups displayed significant improvements in FEV_1,_ FEV_1_%, FVC and FEV_1_/FVC after treatment, and the improvement degree was higher in the observation group than in the control group (*P* < 0.05). After treatment, a higher clinical efficacy was detected in the observation group compared with the control group (*P* < 0.05), but no significant difference was observed in terms of incidence of adverse (*P* > 0.05). LABA/long-acting muscarinic antagonist (LAMA)/ICS triple therapy can effectively improve the pulmonary function and clinical symptoms of patients with stable COPD without increasing adverse drug reactions, consistent with the results of several foreign studies.

COPD refers to a respiratory disease with persistent respiratory airflow limitation; this condition can gradually develop into pulmonary heart disease.^13^,^14^ In severe cases, this disease can lead to respiratory and heart failure, threatening the life safety of patients. As a result, COPD has become one of the most intractable public health problems in the world.^15^,^16^ To date, the pathogenesis of COPD. With an ageing population, declining air quality and an increasing smoking population, COPD is one of the chronic diseases with increased morbidity, disability and mortality rate.^17^ However, this disease is a clinically preventable and treatable disease. Effective treatment measures enable patients with COPD to control disease progression and improve their quality of life. Currently, symptomatic treatments, such as antiasthmatic, bronchial dilation and anti-infection, are preferred clinically for the treatment of COPD. Despite being commonly used in the treatment of COPD, short-acting β2 agonists alone are less effective than expected and difficult to completely clear respiratory secretions.

According to the 2022 edition of the GOLD^18^, upgrading to triple therapy is recommended for patients with COPD who experience acute exacerbations, persistent dyspnoea, limited movement or exacerbations following treatment with ICS + LABA. GOLD affirmed the efficacy of inhaled triple therapy in the treatment of patients with COPD. Flutemevir (fluticasone furoate, umeclidinium bromide and vilanterol trifenatate powder for inhalation) consists of three drug components, namely ‘fluticasone furoate, umeclidinium bromide and vilanterol trifenatate (LABA/LAMA/ICS)’, is a new triple drug used in the field of COPD treatment. This drug, which characterised by convenience and rapid curative effect, was administered once a day. This triple preparation is the only one that has been observed in prospective studies to considerably reduce all-cause mortality in patients with COPD.^19^,^20^ However, reports on the use of LABA/LAMA/ICS triple therapy in China remain limited.

FEV_1,_ FEV_1_%, FVC and FEV_1_/FVC are sensitive indicators for evaluating the severity of COPD and pulmonary function and serve as the gold standard for the clinical diagnosis of COPD.^21^ Patients with COPD suffer from impaired lung function, which is often accompanied by decreased levels of FEV_1,_ FEV_1_%, FVC and FEV_1_/FVC. Thus, the pulmonary function of patients with COPD can be determined through measurement of the levels of FEV_1,_ FEV_1_%, FVC and FEV_1_/FVC to reflect the severity of the patient’s illness.^22^,^23^

Studies have shown the susceptibility of patients with COPD to abnormal cellular immune function, mainly manifested by the decrease in CD3^+^ and CD4^+^ cells and the imbalanced CD4^+^/CD8^+^ ratio. In this study, the two groups showed no significant difference in the levels of CD3^+^, CD4^+^, CD8^+^ and CD4^+^/CD8^+^ before treatment (*P* > 0.05). After treatment, the levels of CD3^+^, CD4^+^, CD8^+^ and CD4^+^/CD8^+^ in the two groups were better than those observed before treatment, and the observation group showed a higher increase degree than the control group (*P* < 0.05), which was is with that reported in the literature.

Factors involved in the progression of COPD and the deterioration of pulmonary function, among which the theory of inflammatory factors has been recognised by many scholars, are incomplete.^24^,^25^ The inflammatory factor TNF-α is mainly produced by monocytes and macrophages and is the earliest and most important major endogenous mediator released in the inflammatory response.^26^ By contrast, LTB4 serves as an important mediator in the pathogenesis of COPD; it is also a potent inflammatory transmitter secreted by lymphocytes, macrophages, neutrophils and other inflammatory cells. LTB4 participates in the aggregation and activation of airway inflammatory cells after acting on the bronchus. This molecule also promotes the contraction of bronchial wall smooth muscle, increases vascular permeability and accelerates glandular secretion, which results in increased airway mucus, mucosal oedema and aggravated COPD inflammatory response.^27^

The imbalance of Th1/Th2 cells has a close relation to the occurrence and development of COPD. IL6 is mainly secreted and produced by Th2 cells, which are closely related to airflow limitation in patients with COPD. Clinically, the level of IL6 serves as the primary indicator in the measurement of the imbalanced Thl/Th2 cells in patients with COPD.^28^ In this study, the two groups showed significantly lower levels of TNF-α, LTB4 and IL-6 than those before treatment, and the observation group presented a greater degree of reduction than the radiation group (*P* < 0.05), demonstrating the satisfactory anti-inflammatory effect of LABA/LAMA/ICS triple therapy. Specifically, LABA/LAMA/ICS results in remarkable inhibition of the synthesis and release of inflammatory cytokines, such as TNF-α, LTB4 and IL-6, and reducing airway inflammatory secretions in patients with COPD. The results revealed significantly reduced SGRQ scores of the two groups after treatment and a higher degree of reduction in the observation group than in the control group (*P*< 0.05), indicating that LABA/LAMA/ICS triple therapy can improve the cardiopulmonary function and daily living ability of patients.

### Limitations:

In this study, the SGRQ scale was used in the comprehensive evaluation of the improvement effect of futimeiwei (fluticasone furoate, umeclidinium bromide and vilanterol trifenatate powder for inhalation) on the quality of life of patients with COPD from the aspects of patient symptoms, activity and impact on daily activities.

## CONCLUSIONS

LABA/LAMA/ICS triple therapy improves the clinical efficacy of the treatment of COPD by lowering the level of inflammatory markers, improving lung function and ameliorating clinical symptoms. This study had a small sample size. Future clinical studies should include more patients to further objectively evaluate the advantages and disadvantages of LABA/LAMA/ICS triple therapy in the treatment of COPD.

### Authors’ Contributions:

**GD and HW:** Carried out the study, participated in collecting data, drafted the manuscript, are responsible and accountable for the accuracy or integrity of the work.

**HW:** Participated in acquisition, analysis, and interpretation of data and drafting the manuscript. All authors read and approved the final manuscript.

## References

[1] Borsi H, Nia EP, Mal-Amir MD, Raji H (2019). Relationship between serum procalcitonin level and chronic obstructive pulmonary disease. J Family Med Prim Care.

[2] Tousa S, Semitekolou M, Morianos I, Banos A, Trochoutsou AI, Brodie TM (2017). Activin-A co-opts IRF4 and AhR signaling to induce human regulatory T cells that restrain asthmatic responses. Proc Natl Acad Sci U S A.

[3] Wang C, Xu J, Yang L, Xu Y, Zhang X, Bai C (2018). Prevalence and risk factors of chronic obstructive pulmonary disease in China (the China Pulmonary Health (CPH) study):a national cross-sectional study. Lancet.

[4] Li H, Shi K, Zhao Y, Du J, Hu D, Liu Z (2020). TIMP-1 and MMP-9 expressions in COPD patients complicated with spontaneous pneumothorax and their correlations with treatment outcomes. Pak J Med Sci.

[5] Jain S, Self WH, Wunderink RG, Fakhran S, Balk R, Bramley AM (2015). Community-Acquired Pneumonia Requiring Hospitalization among U. S. Adults. N Engl J Med.

[6] Asif S, Rasheed A, Mahmud TE, Asghar A (2019). Frequency and predictors of pulmonary hypertension in patients with Systemic Lupus Erythematosus. Pak J Med Sci.

[7] Papi A, Vestbo J, Fabbri L, Corradi M, Prunier H, Cohuet G (2018). Extrafine inhaled triple therapy versus dual bronchodilator therapy in chronic obstructive pulmonary disease (TRIBUTE):a double-blind, parallel group, randomised controlled trial [published correction appears in Lancet.). Lancet.

[8] Shafuddin E, Chang CL, Cooray M, Tuffery CM, Hopping SJ, Sullivan GD (2018). Changes in biomarkers of cardiac dysfunction during exacerbations of chronic obstructive pulmonary disease. Respir Med.

[9] Lee YS, Oh JY, Min KH, Lee SY, Kang KH, Shim JJ (2019). The association between living below the relative poverty line and the prevalence of chronic obstructive pulmonary disease. J Thorac Dis.

[10] Zhou J, Jin F, Wu F (2021). Clinical significance of changes in serum inflammatory factors in patients with chronic obstructive pulmonary disease and pulmonary infection. J Int Med Res.

[11] Adeloye D, Song P, Zhu Y, Campbell H, Sheikh A, Rudan I (2022). Global, regional, and national prevalence of, and risk factors for, chronic obstructive pulmonary disease (COPD) in 2019:a systematic review and modelling analysis. Lancet Respir Med.

[12] Mirza S, Clay RD, Koslow MA, Scanlon PD (2018). COPD Guidelines:A Review of the 2018 GOLD Report. Mayo Clin Proc.

[13] Huang J, Dang F (2022). Analysis of Inducing Factors of Chronic Pulmonary Heart Disease Caused by Chronic Obstructive Pulmonary Disease at High Altitude through Epidemiological Investigation under Intelligent Medicine and Big Data. J Healthc Eng.

[14] You L, Niu H, Huang K, Dong F, Yang T, Wang C (2021). Clinical Features and Outcomes of Acute Exacerbation in Chronic Obstructive Pulmonary Disease Patients with Pulmonary Heart Disease:A Multicenter Observational Study. Int J Chron Obstruct Pulmon Dis.

[15] GBD 2016 Causes of Death Collaborators (2017). Global, regional, and national age-sex specific mortality for 264 causes of death, 1980-2016:a systematic analysis for the Global Burden of Disease Study 2016. Lancet.

[16] Mathers CD, Loncar D (2006). Projections of global mortality and burden of disease from 2002 to 2030. PLoS Med.

[17] Bagdonas E, Raudoniute J, Bruzauskaite I, Aldonyte R (2015). Novel aspects of pathogenesis and regeneration mechanisms in COPD. Int J Chron Obstruct Pulmon Dis.

[18] GOLD:Global strategy for the diagnosis,management and prevention of chronic obstructive pulmonary disease 2022 report.

[19] Lipson DA, Barnhart F, Brealey N, Brooks J, Criner GJ, Day NC, IMPACT Investigators (2018). Once-Daily Single-Inhaler Triple versus Dual Therapy in Patients with COPD. N Engl J Med.

[20] Ismaila AS, Haeussler K, Czira A, Youn JH, Malmenäs M, Risebrough NA (2022). Fluticasone Furoate/Umeclidinium/Vilanterol (FF/UMEC/VI) Triple Therapy Compared with Other Therapies for the Treatment of COPD:A Network Meta-Analysis. Adv Ther.

[21] Laucho-Contreras ME, Cohen-Todd M (2020). Early diagnosis of COPD:myth or a true perspective. Eur Respir Rev.

[22] Serifoglu I, Ulubay G (2019). The methods other than spirometry in the early diagnosis of COPD. Tuberk Toraks.

[23] Bhatt SP, Balte PP, Schwartz JE, Cassano PA, Couper D, Jacobs DR (2019). Discriminative Accuracy of FEV1:FVC Thresholds for COPD-Related Hospitalization and Mortality. JAMA.

[24] Guo P, Li R, Piao TH, Wang CL, Wu XL, Cai HY (2022). Pathological Mechanism and Targeted Drugs of COPD. Int J Chron Obstruct Pulmon Dis.

[25] Wang Z, Locantore N, Haldar K, Ramsheh MY, Beech AS, Ma W (2021). Inflammatory Endotype-associated Airway Microbiome in Chronic Obstructive Pulmonary Disease Clinical Stability and Exacerbations:A Multicohort Longitudinal Analysis. Am J Respir Crit Care Med.

[26] Matera MG, Calzetta L, Cazzola M (2010). TNF-alpha inhibitors in asthma and COPD:we must not throw the baby out with the bath water. Pulm Pharmacol Ther.

[27] Dong R, Xie L, Zhao K, Zhang Q, Zhou M, He P (2015). Cigarette smoke-induced lung inflammation in COPD mediated via LTB4/BLT1/SOCS1 pathway. Int J Chron Obstruct Pulmon Dis.

[28] Mou XF, Tian YP, Guo GH (2005). Implication of interleukin-8, interleukin-6 and c-reactive protein on airway inflammation in chronic obstructive pulmonary disease. Chin J of Nosoc.

